# PLA-Based Hybrid Biocomposites: Effects of Fiber Type, Fiber Content, and Annealing on Thermal and Mechanical Properties

**DOI:** 10.3390/polym15204106

**Published:** 2023-10-16

**Authors:** Supitcha Yaisun, Tatiya Trongsatitkul

**Affiliations:** 1 School of Polymer Engineering, Institute of Engineering, Suranaree University of Technology, Nakhon Ratchasima 30000, Thailand; supitcha_yaisun@hotmail.com; 2 Center for Excellence on Petrochemical and Materials Technology, Chulalongkorn University, Bangkok 10330, Thailand; 3 Research Center for Biocomposite Materials for Medical Industry and Agricultural and Food Industry, Suranaree University of Technology, Nakhon Ratchasima 30000, Thailand

**Keywords:** polylactic acid, coir fiber, bamboo leaf fiber, hybrid composites, annealing, biocomposite

## Abstract

In this study, we utilized a hybridization approach for two different fibers to overcome the drawbacks of single-fiber-reinforced PLA composites. Coir fiber and bamboo leaf fiber were used as reinforcing natural fibers as their properties complement one another. Additionally, we combined thermal annealing with hybridization techniques to further improve the overall properties of the composites. The results showed that the hybridization of BF: CF with a ratio of 1:2 gave PLA-based hybrid composites optimal mechanical and thermal properties. Furthermore, the improvement in the thermal stability of hybrid composites, attributable to an increase in crystallinity, was a result of thermal annealing. The improvement in HDT in annealed 1BF:2CF hybrid composite was about 13.76% higher than that of the neat PLA. Annealing of the composites led to increased crystallinity, which was confirmed using differential scanning calorimetry (DSC). The synergistic effect of hybridization and annealing, leading to the improvement in the thermal properties, opened up the possibilities for the use of PLA-based composites. In this study, we demonstrated that a combined technique can be utilized as a strategy for improving the properties of 100% biocomposites and help overcome some limitations of the use of PLA in many applications.

## 1. Introduction

Biopolymers are presently viewed as promising substitutes for traditional petroleum-based polymers, as the latter have contributed to environmental issues related to pollution, greenhouse gas emissions, and the depletion of fossil fuel reserves. Growing environmental awareness and sustainability concerns among consumers have driven industries to search for alternative, more environmentally friendly materials [1]. Polylactic acid or PLA stands out as one of the extensively studied and commonly used biopolymers, garnering significant interest for traditional uses like packaging materials, fiber production, and more recently, in composite materials for diverse practical and mechanical applications. PLA occupies a central role in the eco-friendly polymer market and emerges as a highly promising choice for future advancements [2,3,4]. However, the use of PLA has been limited by its thermal properties. It has a low heat-softening temperature and low thermal stability as compared with petroleum-based polymers polyethylene (HDPE), polypropylene (PP), and polystyrene (PS).

To overcome these limitations while maintaining a status of being 100% biodegradable, natural fibers used as reinforcement in PLA-based biocomposites have been investigated to improve PLA performance [5]. An increasing tendency has been observed in favor of incorporating natural fibers as a reinforcement in polymer composites. This inclination is driven by their adaptability during processing, well-defined strength characteristics, ready availability, biodegradable nature, cost-effectiveness (in terms of volume), and environmentally friendly attributes. Polymer composites incorporating natural fibers present numerous notable benefits compared with traditional synthetic alternatives, including their biodegradability, environmentally friendly characteristics, affordability, ready availability, low weight, and more. These natural fiber-reinforced polymer composites are receiving increasing recognition and wider acceptance across various applications, such as food packaging, interiors of automobiles, railway coaches, and airplanes, as well as storage solutions and construction.

Recently, coir fibers (CFs) have stood out as excellent choices due to the fruit’s remarkable versatility, serving a purpose as a food and contributing to the production of a diverse array of industrial products. The extensive cultivation of coir crops results in substantial biomass accumulation, often leading to its disposal in landfills or inappropriate sites, giving rise to significant social and environmental challenges. On the other hand, coir fiber-reinforced polylactic acid (PLA) biocomposites have gained substantial research attention for their use in various applications [6,7,8,9,10]. Coir fibers possess low cellulose (36–43%) and hemicellulose (0.2%), a high lignin content (41–45%), and a high microfibrillar angle (30–45°, which results in their relatively low tensile strength and modulus as well as the highest elongation at the break among other typical natural fibers [11,12,13], as shown in Table 1. Because coir fibers possess a significant amount of lignin, they exhibit durability, resistance to weather, a degree of waterproofing, and the potential for chemical modification. Additionally, these fibers can be stretched beyond their elastic limit without breaking, showcasing a high elongation at the break [14]. Many studies in the literature contain comprehensive analyses of the structural, morphological, mechanical, and thermal characteristics of coir fibers [15,16,17,18,19,20]. Bamboo leaf fiber (BF) is one of the most abundantly available waste materials. BF has a shorter growing time than its culm. Studies on the use of BF as a reinforcement material have not yet been widespread. Up to now, studies have indicated that adding fibers into PLA matrices can improve the performance of PLA composites. The selection of plant fibers determines the end properties of composites [21].

Incorporating natural fibers into polymer composites can be challenging due to certain inherent characteristics that have potential downsides. These characteristics include limited protection against microbial attacks, inadequate resistance to moisture, poor adhesion in the fiber–matrix surface, and a tendency to form aggregates during processing. These limitations can be addressed by alterations to the surface of fibers; this is achievable using chemical techniques like mercerization [22], dewaxing, acetylation, chemical grafting, bleaching, delignification, and salinization [23].

Because each fiber possesses unique advantages and drawbacks, reinforcing a given polymer with a combination of two or more fiber types may help to overcome the drawbacks of each fiber and, subsequently, improve the properties of the composite overall. This technique is known as “hybrid composite” and has recently attracted significant attention from researchers [9,24,25].

To further improve the mechanical properties and service temperatures of PLA-based composites, we used thermal annealing together with hybridization in this study. Thermal annealing can be carried out by subjecting specimens to a high temperature, above its cold-crystallization temperature, and then using a slow cooling rate to induce the formation of a crystallized structure that enhances the thermal properties of the material, as well as the mechanical properties [26,27,28]. Thus, the service temperature and mechanical performance of the PLA-based composite are expected to improve.

Many researchers try to improve natural fiber composite properties by carrying out chemical treatment of the natural fiber [29]. However, because most of the chemical treatment techniques use strong acidic or basic chemicals, they are inherently harmful to the environment. Therefore, we focused on developing a 100% eco-friendly material in this work. We strategically combined fiber hybridization and annealing to overcome the drawbacks of PLA composites. In this study, first, we investigated the effect of fiber types (coir and bamboo leaf fibers), fiber loading (5, 10, and 15 wt%), and thermal annealing on morphology, tensile properties, thermal properties, crystallinity, and heat distortion temperature (HDT) of a PLA-based single-fiber composite. Then, we combined the fiber in various ratios to create PLA-based hybrid composites. The fiber loading of 10 wt% was kept constant. The ratio of coir fiber and bamboo leaf fiber was varied. The BF: CF ratios were 1:1, 1:2, and 2:1. The fibers were incorporated into the PLA matrix using twin screw extrusion, and the test specimens were prepared with compression molding. To enhance the crystallization of PLA, annealing of PLA-based hybrid composites was performed at 120 °C for 30 min [30]. With these combined techniques, an improvement in the overall properties of composites could be expected.

**Table 1:** The chemical, mechanical, and physical properties of natural fibers [31].

Sample	Coir	Bamboo	Bamboo Leaf	Sisal
Density (g/m^3^)	1.25–1.5	0.9	-	1.26–1.33
Diameter (µm)	100–450	-	-	100–300
Cellulose (%)	36–43	26–43	19.5–26.3	74–75.2
Hemicellulose (%)	0.2	30	11.3–13.5	10–13.9
Lignin (%)	41–45	21–30	8.7–11.6	8–12
Microfibrillar angle (°)	30–45	-	-	10–20
Tensile strength (MPa)	105–175	-	-	600–700
Young’s modulus (GPa)	4–6	-	-	38
Elongation at break (%)	17–47	-	-	3.64–5.12
Moisture absorption (%)	10	-	-	11

## 2. Materials and Methods

### 2.1. Materials

Polylactic acid (PLA) (grade LX175, Purac Ltd., Ban Chang, Thailand) used was at an extrusion grade with a density of 1.24 g/cm^3^ and a melt flow index of 3 g/10 min. Coir (*Cocos nucifera* L., Thailand) and bamboo leaf (*Bambusa ventricosa McClure*, Thailand) were purchased from local farmers in Nakhon Ratchasima, Thailand.

#### 2.1.1. Fiber Preparation

The dried bamboo leaf and coir were crushed into fine fibers with shorter lengths using a wood crusher machine (CT, CGR-20, Chareon Tut Co., Ltd., Samutprakarn, Thailand) for 1 h. The fibers with a diameter in the range of 45–106 µm were obtained. The obtained fibers were given code names as bamboo leaf fiber (BF) and coir fiber (CF).

#### 2.1.2. Preparation of PLA-Based Composites

**Figure 1:**
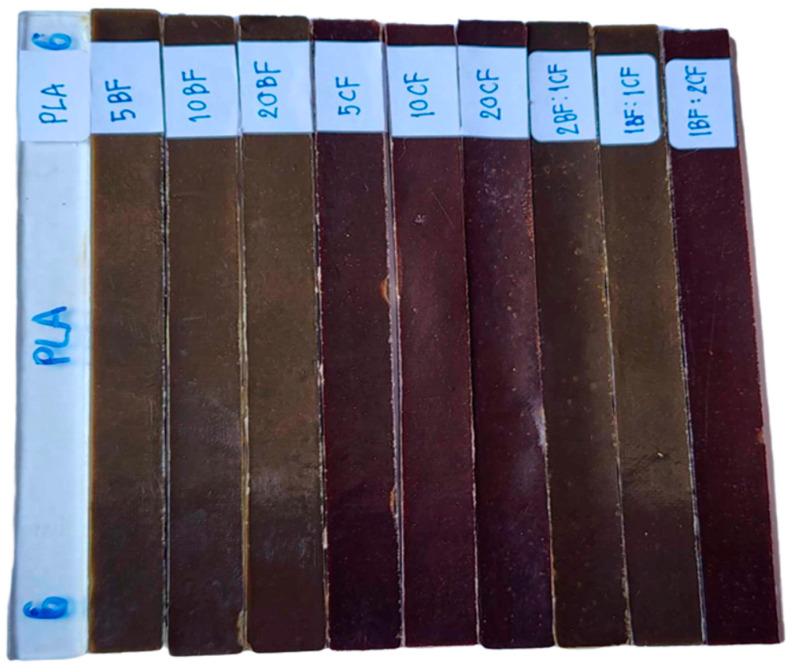
Photograph of PLA, PLA composite, and PLA-based hybrid composite specimens.

A list of samples used in this study is shown in Table 2. The composites were prepared with the melt mixing technique using a co-rotating twin screw extruder (Brabender, DSE 35/17D, Brabender GmbH & Co. KG, Duisburg, Germany). The fibers and PLA were dried in a hot air oven at 80 °C for 4 h before use. Immediately after drying, the PLA and fiber underwent melt mixing in the twin screw extruder at the screw speed of 20 rpm and melting temperature of 170 °C. The compound pellets were then compression molded at 170 °C for 10 min to form test specimens (See Figure 1). To investigate the effect of annealing on the composite’s properties, samples were annealed at 120 °C for 30 min in a hot air oven (Despatch, LAC series, Despatch Industries, Inc., Lakeville, MN, USA) before being left cool at room temperature.

### 2.2. Characterization and Test

#### 2.2.1. Tensile Test

The tensile test of PLA and its composite was carried out according to ASTM D638 [32]. Five dog bone-shape specimens with a gauge length of 50 mm were tested at room temperature (~25 °C) using a universal testing machine (UTM, INSTRON/5565, Instron Co., Ltd., Norwood, MA, USA). The test was performed using a 5 kN load cell at 5 mm/min crosshead speed. The reported value is an average value from five replications. The error bars shown in the graph represent the standard deviation value.

#### 2.2.2. Morphological Study

The tensile fractured surfaces of PLA and its composites were used in the investigation of the composites’ morphological structure. The fractured surface was used as it can reveal information on the distribution and dispersion of the reinforcing agents and the adhesion between the fibers and matrix as well as the failure mode (brittle or ductile fracture). The fractured surfaces were then sputtered coated with gold for 3 min before being examined using a scanning electron microscope, SEM (JEOL, model JSM6400, JEOL Ltd., Tokyo, Japan), at 5–10 kV.

#### 2.2.3. Differential Scanning Calorimetry (DSC)

The crystallization and melting behaviors of PLA and PLA-based hybrid composites were determined using differential scanning calorimetry (DSC: Mettler Toledo STARe SW 8.1, Mettler-Toledo International Inc., Greifensee, Switzerland). A sample was heated from 25 to 200 °C with a heating rate of 10 °C/min (first heating scan). After keeping the sample at 200 °C for 1 min, it was cooled to 25 °C. Finally, it was heated again to 200 °C (second heating scan). The degree of crystallinity ($X_{c}$) of the neat PLA, PLA composites, and PLA hybrid composites was calculated using Equation (1) [33].

(1) $$X_{c}=\frac{\Delta H_{m}-\Delta H_{cc}}{\omega \Delta H_{m}^{o}}\times 100\%$$

where $\Delta H_{m}$ is the heat of melting and $\Delta H_{cc}$ is determined by integrating the areas (J/g) under the peaks. $\Delta H_{m}^{o}$ is a reference value and represents the heat of melting if the polymer were 100% crystallinity (93.7 J/g for PLA) [34] and ω is the weight fraction of the PLA in the composites.

### 2.3. Heat Deflection Temperature (HDT)

An HDT/VICAT manual heat deflection tester (model HDV1, Atlas Electric Devices Co., Chicago, IL, USA) was used to measure the heat deflection temperature (HDT) of PLA and its composites. The test was carried out using a load of 0.455 MPa, as specified by ASTM D648 [35].

## 3. Results and Discussion

### 3.1. Effect of Fiber Type and Content on Tensile Properties of PLA-Based Composites

**Figure 2:**
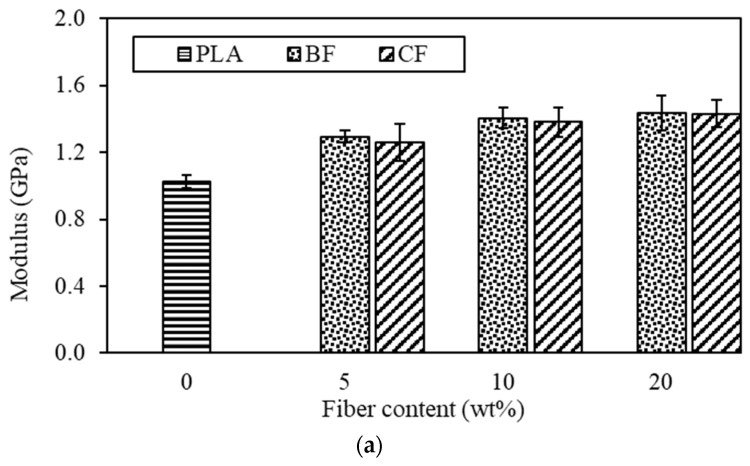

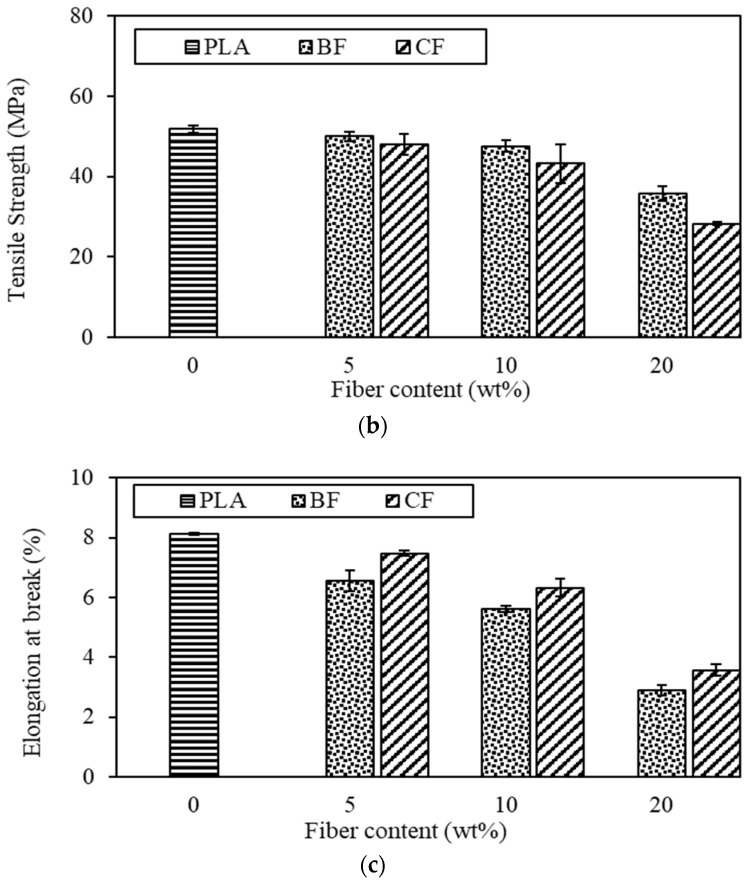
Tensile properties of PLA and PLA composites with varied fiber content 5–20 wt% (**a**) tensile modulus, (**b**) tensile strength, and (**c**) elongation at break.

The tensile properties including modulus tensile strength, and elongation at break of PLA and PLA single-fiber composites at various fiber loading are depicted in Figure 2. Generally, neat PLA showed better tensile strength and elongation at break than those of the PLA composites. This finding is in agreement with those reported by others [36,37]. The neat PLA possessed the highest tensile strength and elongation at break of 51.85 MPa and 8.13%, respectively. The presence of natural fiber in PLA composite increased the tensile modulus. The maximum tensile moduli were obtained from the composites containing 20 wt% fiber loading. Improvements in tensile moduli in the PLA composites over the neat PLA were 41.18% and 40.20% for 20BF/PLA and 20CF/PLA, respectively.

Increasing the fiber loading resulted in an increase in the tensile moduli of PLA composites, whereas the tensile strength and elongation at break of PLA composites were decreased. The results suggested that with the presence of the natural fiber, the PLA-based composite became more brittle. These results were expected, and they indicated a poor adhesion between the natural fiber and the PLA matrix. Similar results were reported by other researchers who suggested that these findings were associated with a low strength of adhesion among the fibers within composite materials. The decrease in tensile strength with the increasing fiber content may also be due to an agglomeration of fibers in the PLA matrix [36,37,38]. The poor adhesion among the composite components stemmed from the low hydrophilicity and polarity of PLA as compared with those of the plant fibers, which possess polar hydroxyl groups on their surfaces. The PLA composites reinforced with plant fibers tend to display inadequate interfacial adhesion, consequently leading to ineffective stress transfer from the matrix to the fibers [39,40,41]. A poor adhesion between the fibers and the PLA matrix could be seen in the results of the morphological study reported in the next section. In addition, it was known that adding rigid particles into a polymer matrix generally resulted in an increased stiffness of the polymer.

As compared with the same fiber content, the presence of BF and CF in the PLA matrix gave the same moduli value, which increased with the fiber loading. Therefore, the type of fiber was an insignificant parameter in increasing the composite stiffness. In other words, the fiber content played a dominant role in controlling the composite moduli. On the other hand, the type of fiber seemed to have a significant effect on the tensile strength and elongation at break. The BF/PLA composites showed slightly higher tensile strength, while the CF/PLA composites presented higher elongation at break. These findings could plausibly be explained by the nature of the fiber (see Table 1). The coir fiber possessed a low tensile strength and high stretching ability as compared with the other fibers. Therefore, a composite of such fiber would sustain the same characteristics as its component [11,12,13,14]. Additionally, one could speculate that combining the two fibers would yield the best characteristic of both fibers. To investigate the effect of fiber hybridization, we considered using 10 wt% fiber for further study. Although the PLA composite containing 20 wt% of fiber possessed the highest modulus, because of the diminishing tensile strength and elongation at break, it may not be optimal for further improvement in the composite properties. PLA composites with 10 wt% fiber content possessed overall optimal properties and offered the possibility for greater improvement as well as the opportunity to understand the different effects of different fibers in hybridization on the composite performance. Therefore, hybrid composites with different ratios of BF: CF were prepared with a constant fiber loading of 10 wt%.

### 3.2. Effect of Fiber Ratio and Annealing on Tensile Properties of PLA-Based Composites

**Figure 3:**
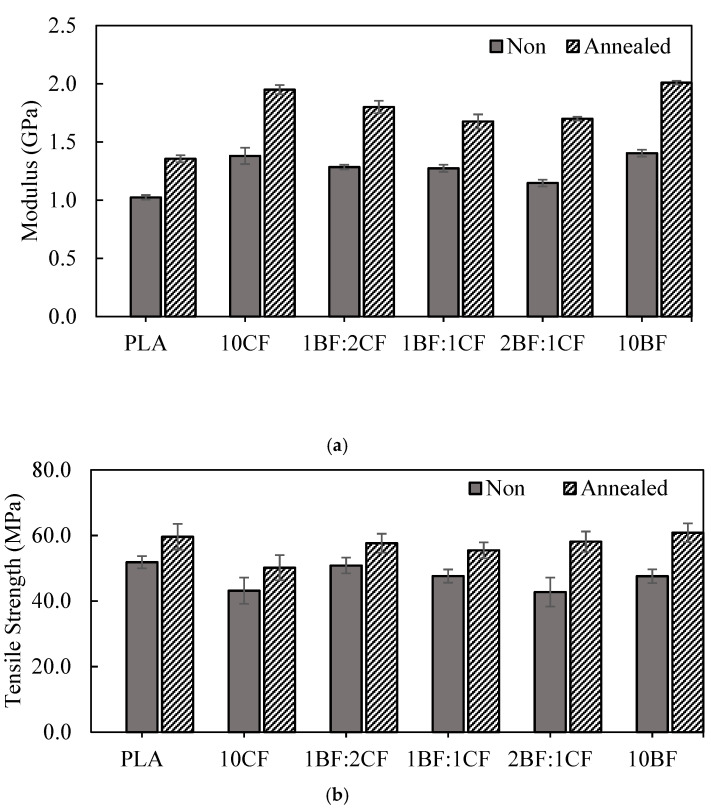

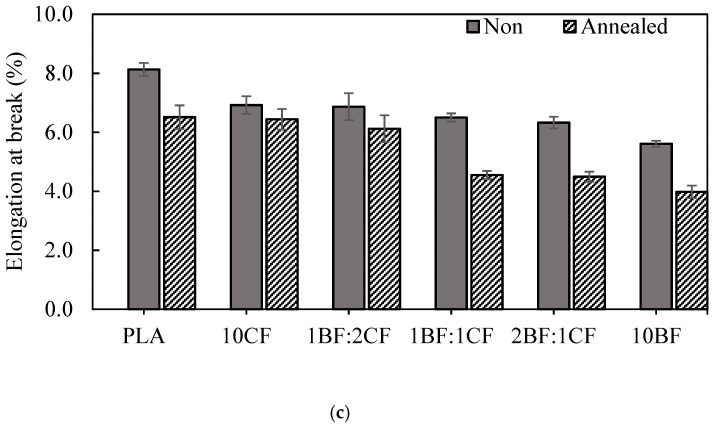
Tensile properties of PLA, PLA composites, and PLA hybrid composites with and without thermal treatment (annealing at 120 °C for 30 min): (**a**) tensile modulus, (**b**) tensile strength, and (**c**) elongation at break.

Hybrid composites with a constant fiber loading of 10 wt% were prepared with different BF: CF ratios (1:1, 1:2, and 2:1). As can be seen in Figure 3, similar to the results found for single-fiber composites, the modulus of the hybrid composites was dependent only on the fiber loading. Different BF: CF fiber ratios gave insignificantly different modulus as well as tensile strength. Among the hybrid composites, 1BF: 2CF showed the highest tensile properties including tensile modulus, tensile strength, and elongation at break. It was interesting to note that elongation at break of the composites seemed to improve with the CF fiber content. As the ratio of the reinforced fiber changed, the decrease in CF content in the composite resulted in a decrease in elongation at break. This finding that CF was beneficial in improving elongation at break of single-fiber-reinforced PLA remained true even in the hybrid composite, where two different fibers were combined.

Annealing was the strategy we used in combination with fiber hybridization. The increases in modulus and tensile strength were expected as annealing generally increased and improved crystallinity and crystal growth. The crystalline phase in a semicrystalline polymer is known to be the main contributor to the hardening and strengthening of the polymer. As expected, further increases in modulus and tensile strength were obtained after thermal treatment/annealing of the PLA hybrid composites. However, elongation at break of all annealed samples suffered, as shown in Figure 3. These could also be explained by the increase in crystallinity in post-annealing samples. Higher crystalline materials resulted in harder material, whereas the portion of amorphous regions providing the elasticity of the sample was reduced. The presence of an increased crystal portion restricted the chain movement in the samples, resulting in lower extensibility. The DSC results reported in the next section confirm this speculation. It should be noted that the hybrid composite containing a high content of coir fiber showed a lower degree of elongation at break dimension after annealing. The decrease in elongation at break was about 7% and 10% in 10CF and 1:2 BF: CF composites, respectively, and about 30% for the other composites. This result indicated the strong dependency of elongation at break on fiber type even after annealing, where crystallinity should be dominant. To our knowledge, this particular point has not been reported elsewhere and may be worth investigating in depth in future studies.

### 3.3. Morphology of PLA-Based Composites

**Figure 4:**
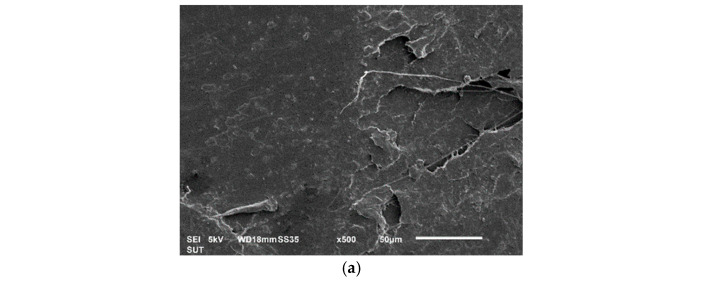

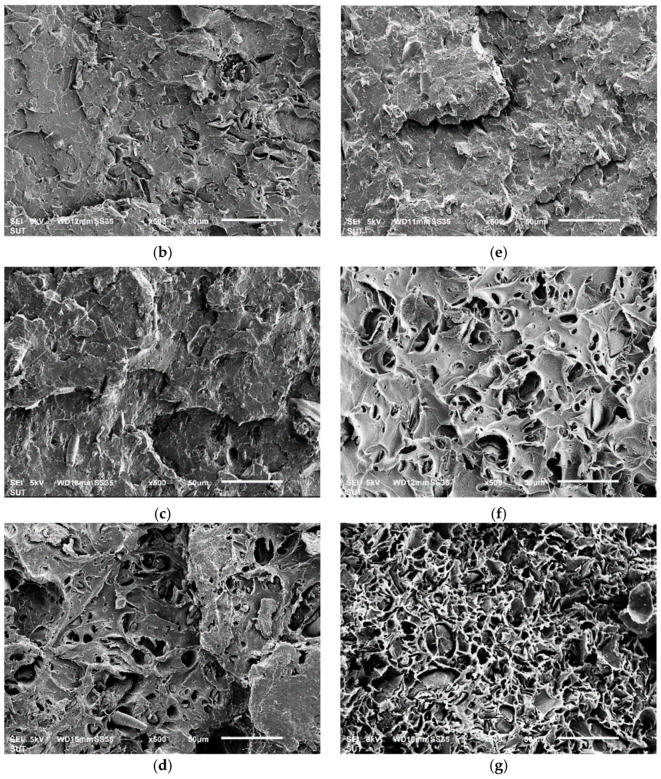
SEM micrographs of the tension-fractured surfaces of (**a**) neat PLA, (**b**) 5BF/PLA, (**c**) 10BF/PLA, (**d**) 20BF/PLA, (**e**) 5CF/PLA, (**f**) 10CF/PLA, and (**g**) 20 CF/PLA.

SEM micrographs of the tensile fractured surface of PLA and its composites are shown in Figure 4. The surface of neat PLA was smooth, all the composites were present with voids. Figure 4b–g shows the void due to fiber pull-out and poor adhesion between the fibers and matrix [42]. The void size tends to increase with increasing fiber content. Composites with CF showed more voids with greater sizes than those with BF. These voids generate weak zones where the load-bearing capacity tends to drop, leading to a reduction in tensile strength and elongation at break of PLA composites. When comparing BF composites and CF composites, the fractured surface investigation revealed that CF composites were more ductile as compared with the BF composites. The yielding feature of the PLA matrix on the fractured surface of CF composites was plausibly due to the ability of CF to elongate to a greater extent than BF [14]. Thus, when the extensional force was applied, the CF held the composites together, impeding a brittle failure and allowing a greater extension length before braking. This finding agreed well with the tensile results shown in Figure 2. The results also suggested that CF may have a better adhesion between fibers and the PLA matrix; otherwise, fracture surface yield could not occur.

### 3.4. Thermal and Crystallinity of PLA-Based Composites

**Figure 5:**
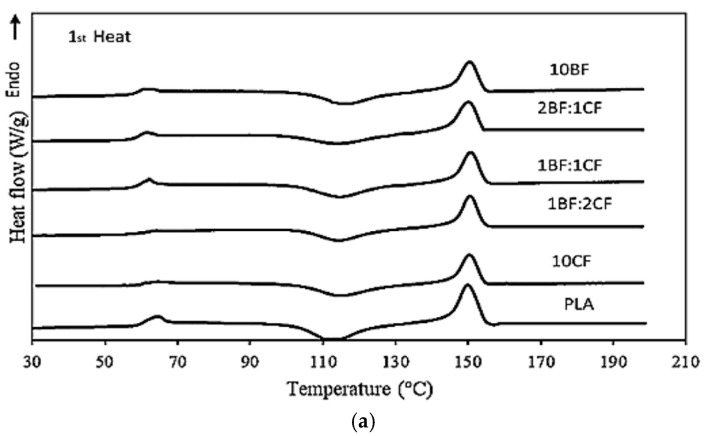

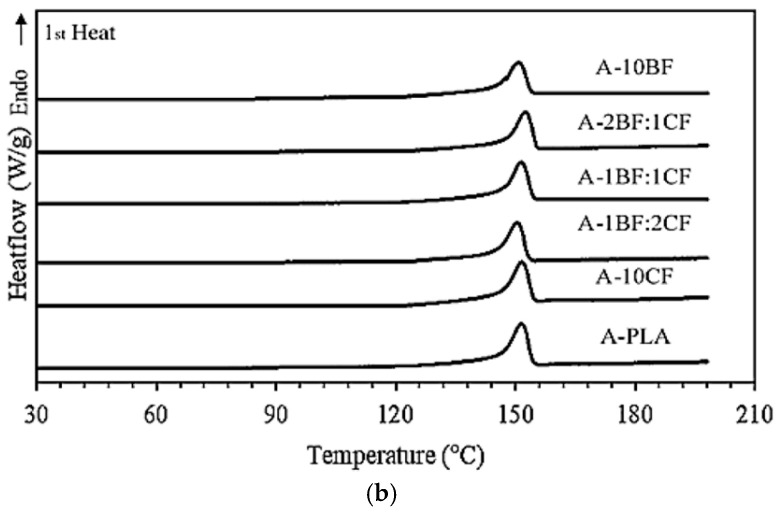
DSC thermograms of PLA, PLA composites, PLA hybrid composites (**a**) before and (**b**) after heat treatment.

Differential scanning calorimetry (DSC) was used to investigate the effect of fiber type, fiber content, and thermal treatment in promoting the crystallinity of the PLA matrix. The DSC data including crystallinity (Xc) calculated using Equation (1), melting temperature (T_m_), and cold crystallization temperature (T_cc_) from the first heating scan are presented in Figure 5 and Table 3. It can be seen that the T_g_ of PLA was 61.18 °C. The T_g_ slightly decreased with the addition of fiber into the PLA matrix. The T_CC_ was observed in all non-annealed samples, indicating that PLA molecules were unable to crystallize during the cooling phase. It is well-known that PLA’s crystal formation is naturally low and requires significant encouragement to induce crystallization [43]. An increase in crystallinity in PLA results in an improvement in several properties such as tensile and thermal properties [44]. T_cc_ of PLA composites increased with the presence of fiber. This indicated a greater amount of the non-crystalline phase in the composite as compared with that of the neat PLA. The result agreed well with the crystallinity (*X_C_*). This was plausibly due to the incorporated fiber restricting the mobility of PLA chains together with the fast-cooling conditions during compression molding [45]. However, the T_g_ and T_cc_ peaks disappeared from the curve after thermal annealing. The disappearance of the peaks signified the growth of crystals in PLA (crystal perfection). This phenomenon was attributed to the rearrangement of PLA molecules upon high temperature and slow cooling. The molecules had sufficient time to slowly crystallize.

The $X_{C}$ of the neat, non-annealed PLA was 6.1%, which showed the tendency to decrease with increasing fiber content. The nucleating ability of both fillers was insufficient to obtain the dominant crystallinity of the PLA-based composites. The existence of both fibers hindered the mobility of PLA chains, leading to the poor rearrangement of PLA molecules and thus, lower crystallinity. The higher results for $X_{C}$ of post-annealed samples were due to sufficient time and energy for the PLA molecules to rearrange and overcome the hindrance of the fiber to crystallization during the annealing process.

### 3.5. Heat Deflection Temperature (HDT)

**Figure 6:**
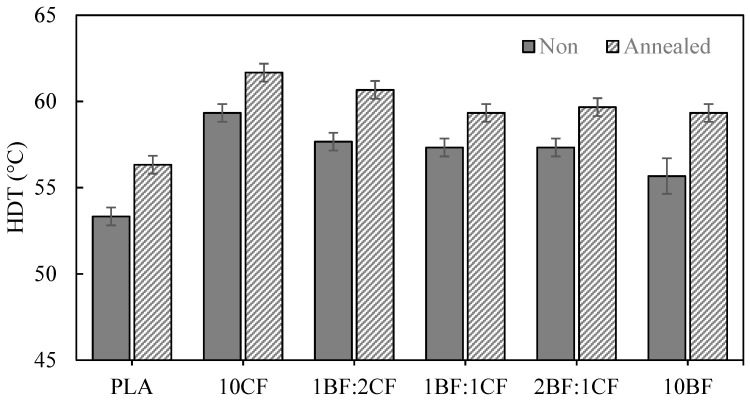
HDT data for PLA, PLA composites, and PLA hybrid composites before and after heat treatment.

The heat deflection temperature (HDT) is commonly used to determine the maximum service temperature of a material. PLA possesses a low service temperature, which limits its use in various applications. For semicrystalline polymers such as PLA, HDT is strongly dependent on crystallinity. The poor HDT of PLA is partly due to its naturally low crystallinity. Figure 6 shows the HDT results of PLA and its composites. The HDT of neat PLA was about 53.33 °C, which was rather low for various applications such as automotive parts and packaging. With the presence of natural fiber in the PLA matrix, the HDT increased to 55.67 and 59.33 °C for the 10BF/PLA and 10CF/PLA composites, respectively. The increase in HDT in this case was due to the higher stiffness (moduli) of the single-fiber composites. As discussed previously, the presence of fiber in the composites reduced the crystallinity slightly; therefore, the increase in HDT in the composite was not from the crystallinity.

In the case of hybrid composites created by adding BF: CF in different ratios (1BF:2CF, 1BF:1CF, and 2BF:1CF) into the PLA matrix, the HDT value of 1BF:2CF was the highest among those hybrid composites. The HDT value of 2BF:1CF was 57.33 °C, which was a 7.5% improvement as compared with the neat PLA. The increases in HDT for PLA hybrid composites were also due to the increase in stiffness with the presence of fiber and not because of crystallinity. Stiffness is defined as the ability of a material to resist deformation under load. On the other hand, HDT is a measurement of the material while temperature is increased. Therefore, the modulus of PLA composites and PLA hybrid composites were also examined for an analysis of HDT. Referring to Figure 2a and Figure 3a, it was shown that the modulus of PLA composites and PLA hybrid composites were higher than that of the neat PLA.

Annealing of the PLA composites gave rise to HDT in all samples. This was because annealing increased both the crystallinity and modulus. The increase in HDT value of the post-annealing samples might be due to the increase in crystallinity and consequently, the modulus/stiffness. The high-temperature exposure and slow cooling process of PLA composites induce the formation of a crystallized structure that enhances thermal properties. The maximum HDT obtained in this work was 61.7 °C in an annealed 10CF sample, which was an 8.3 °C increase over the non-annealed neat PLA. Other researchers also reported an increase in HDT over the same temperature range. A further increase in HDT up to 120 °C could be achieved using nucleating agents together with thermal treatment manipulation [46].

## 4. Conclusions

In summary, we illustrated the possibility of improving PLA properties using combined techniques of hybridization and thermal treatment. The hybrid composites created using two different fibers can offer beneficial properties of the individual fibers used. Selecting a proper pair of fibers is critical to obtaining properties that complement one another. In this work, the PLA composite reinforced with coir fiber offers better elongation at break and HDT than BF composites, while bamboo leaf fiber offers better tensile strength than CF composites. The fiber content played an important role in dominating the mechanical properties of the final composite, specifically the stiffness (moduli). The PLA composites consisting of 10 wt% of fiber possessed the most balanced properties in terms of tensile modulus, tensile strength, and elongation at break. Hybridization of the BF: CF with the ratio of 1:2 gave the most desirable properties. Thermal treatment or annealing further improved the mechanical and thermal properties of hybrid composites. These improvements are attributed to the increased degree of crystallinity brought about by the exposure to high temperatures and slow cooling. However, the result of tensile properties showed lower tensile strength, as compared with the neat PLA, due to poor adhesion between fiber and matrix. Further studies can be performed to investigate several strategies to improve the adhesion between fibers and the PLA matrix. It can be expected that when the adhesion between fibers and the matrix is improved, the properties of PLA-based hybrid composites will be greatly improved, which will consequently open more possibilities for the utilization of PLA in various applications.

## Figures and Tables

**Table 2:** Composition of PLA-based composites.

Sample	PLA (wt%)	BF (wt%)	CF (wt%)
Neat PLA	100	-	-
5BF/PLA	95	5	-
10BF/PLA	90	10	-
20BF/PLA	80	20	-
5CF/PLA	95	-	5
10CF/PLA	90	-	10
20CF/PLA	80	-	20
1BF:2CF	90	3.33	6.67
1BF:1BF	90	5	5
2BF:1CF	90	6.67	3.33

**Table 3:** Transition temperatures obtained from the DSC first heating scan of PLA and its composites, with and without thermal treatment.

Sample	Non-Annealed				Annealed			
	T_g_ (°C)	T_cc_ (°C)	T_m_ (°C)	$X_{C}$ (%)	T_g_ (°C)	T_cc_ (°C)	T_m_ (°C)	$X_{C}$ (%)
PLA	61.18	112.43	149.84	6.10	-	-	151.82	38.90
10CF	61.28	115.22	150.30	2.85	-	-	151.32	36.73
1BF:2CF	59.99	114.20	150.45	4.68	-	-	151.62	37.38
1BF:1CF	59.63	114.53	150.62	3.63	-	-	150.29	36.36
2BF:1CF	59.59	114.04	150.12	2.08	-	-	150.76	36.23
10BF	59.75	116.25	150.47	2.44	-	-	150.47	35.72

## Data Availability

The data presented in this study are available upon request from the corresponding author.

## References

[1] Mohanty A.K., Misra M., Drzal L.T. (2002). Sustainable Bio-Composites from Renewable Resources: Opportunities and Challenges in the Green Materials World. J. Polym. Environ..

[2] Siracusa V., Rocculi P., Romani S., Rosa M.D. (2008). Biodegradable polymers for food packaging: A review. Trends Food Sci. Technol..

[3] Pongtanayut K., Thongpin C., Santawitee O. (2013). The Effect of Rubber on Morphology, Thermal Properties and Mechanical Properties of PLA/NR and PLA/ENR Blends. Energy Procedia.

[4] Yang Y., Zhang L., Xiong Z., Tang Z., Zhang R., Zhu J. (2016). Research progress in the heat resistance, toughening and filling modification of PLA. Sci. China Chem..

[5] Trifol J., Plackett D., Sillard C., Szabo P., Bras J., Daugaard A.E. (2016). Hybrid poly(lactic acid)/nanocellulose/nanoclay composites with synergistically enhanced barrier properties and improved thermomechanical resistance. Polym. Int..

[6] Coskun K., Mutlu A., Dogan M., Bozacı E. (2019). Effect of various enzymatic treatments on the mechanical properties of coir fiber/poly(lactic acid) biocomposites. J. Thermoplast. Compos. Mater..

[7] Dong Y., Ghataura A., Takagi H., Haroosh H.J., Nakagaito A.N., Lau K.-T. (2014). Polylactic acid (PLA) biocomposites reinforced with coir fibres: Evaluation of mechanical performance and multifunctional properties. Compos. Part A Appl. Sci. Manuf..

[8] Sun Z., Zhang L., Liang D., Xiao W., Lin J. (2017). Mechanical and Thermal Properties of PLA Biocomposites Reinforced by Coir Fibers. Int. J. Polym. Sci..

[9] Yusoff R.B., Takagi H., Nakagaito A.N. (2016). Tensile and flexural properties of polylactic acid-based hybrid green composites reinforced by kenaf, bamboo and coir fibers. Ind. Crops Prod..

[10] Zhang L., Sun Z., Liang D., Lin J., Xiao W. (2017). Preparation and performance evaluation of PLA/coir fibre biocomposites. BioResources.

[11] Adeniyi A.G., Onifade D.V., Ighalo J.O., Adeoye A.S. (2019). A review of coir fiber reinforced polymer composites. Compos. Part B Eng..

[12] Mathura N., Cree D. (2016). Characterization and mechanical property of Trinidad coir fibers. J. Appl. Polym. Sci..

[13] Nam T.H., Ogihara S., Tung N.H., Kobayashi S. (2011). Effect of alkali treatment on interfacial and mechanical properties of coir fiber reinforced poly(butylene succinate) biodegradable composites. Compos. Part B Eng..

[14] Geethamma V.G., Kalaprasad G., Groeninckx G., Thomas S. (2005). Dynamic mechanical behavior of short coir fiber reinforced natural rubber composites. Compos. Part A Appl. Sci. Manuf..

[15] Zainudin E.S., Yan L.H., Haniffah W.H., Jawaid M., Alothman O.Y. (2014). Effect of coir fiber loading on mechanical and morphological properties of oil palm fibers reinforced polypropylene composites. Polym. Compos..

[16] Arrakhiz F.Z., El Achaby M., Malha M., Bensalah M., Fassi-Fehri O., Bouhfid R., Benmoussa K., Qaiss A. (2013). Mechanical and thermal properties of natural fibers reinforced polymer composites: Doum/low density polyethylene. Mater. Des..

[17] Essabir H., Bensalah M.O., Rodrigue D., Bouhfid R., Qaiss A. (2016). Structural, mechanical and thermal properties of bio-based hybrid composites from waste coir residues: Fibers and shell particles. Mech. Mater..

[18] Morandim-Giannetti A.A., Agnelli J.A.M., Lanças B.Z., Magnabosco R., Casarin S.A., Bettini S.H.P. (2012). Lignin as additive in polypropylene/coir composites: Thermal, mechanical and morphological properties. Carbohydr. Polym..

[19] Rosa M.F., Chiou B.-S., Medeiros E.S., Wood D.F., Williams T.G., Mattoso L.H.C., Orts W.J., Imam S.H. (2009). Effect of fiber treatments on tensile and thermal properties of starch/ethylene vinyl alcohol copolymers/coir biocomposites. Bioresour. Technol..

[20] Islam M.N., Haque M.M., Huque M.M. (2009). Mechanical and Morphological Properties of Chemically Treated Coir-Filled Polypropylene Composites. Ind. Eng. Chem. Res..

[21] Peltola H., Pääkkönen E., Jetsu P., Heinemann S. (2014). Wood based PLA and PP composites: Effect of fibre type and matrix polymer on fibre morphology, dispersion and composite properties. Compos. Part A Appl. Sci. Manuf..

[22] Suardana N., Lokantara I.P., Lim J.K. (2011). Influence of water absorption on mechanical properties of coconut coir Fiber/Poly-Lactic acid biocomposites. Mater. Phys. Mech..

[23] Saw S.K., Sarkhel G., Choudhury A. (2011). Surface modification of coir fibre involving oxidation of lignins followed by reaction with furfuryl alcohol: Characterization and stability. Appl. Surf. Sci..

[24] Irina M.M.W., Azmi A.I., Tan C.L., Lee C.C., Khalil A.N.M. (2015). Evaluation of Mechanical Properties of Hybrid Fiber Reinforced Polymer Composites and their Architecture. Procedia Manuf..

[25] Sanjay M.R., Yogesha B. (2017). Studies on Natural/Glass Fiber Reinforced Polymer Hybrid Composites: An Evolution. Mater. Today Proc..

[26] Kong W., Zhu B., Su F., Wang Z., Shao C., Wang Y., Liu C., Shen C. (2019). Melting temperature, concentration and cooling rate-dependent nucleating ability of a self-assembly aryl amide nucleator on poly(lactic acid) crystallization. Polymer.

[27] Tábi T., Sajó I.E., Szabó F., Luyt A.S., Kovacs J.G. (2010). Crystalline structure of annealed polylactic acid and its relation to processing. Express Polym. Lett..

[28] Wang J., Kazemi Y., Wang S., Hamidinejad M., Mahmud M.B., Pötschke P., Park C.B. (2020). Enhancing the electrical conductivity of PP/CNT nanocomposites through crystal-induced volume exclusion effect with a slow cooling rate. Compos. Part B Eng..

[29] Bouzouita A., Notta-Cuvier D., Raquez J.-M., Lauro F., Dubois P., Di Lorenzo M., Androsch R. (2017). Poly(lactic acid)-Based Materials for Automotive Applications. Industrial Applications of Poly(Lactic Acid).

[30] Shi Q.F., Mou H.Y., Li Q.Y., Wang J.K., Guo W.H. (2012). Influence of heat treatment on the heat distortion temperature of poly(lactic acid)/bamboo fiber/talc hybrid biocomposites. J. Appl. Polym. Sci..

[31] Nam T.H., Ogihara S., Kobayashi S. (2012). Interfacial, Mechanical and Thermal Properties of Coir Fiber-Reinforced Poly(Lactic Acid) Biodegradable Composites. Adv. Compos. Mater..

[32] (2022). Standard Test Method for Tensile Properties of Plastics.

[33] Choi H.-J., Kim M.S., Ahn D., Yeo S.Y., Lee S. (2019). Electrical percolation threshold of carbon black in a polymer matrix and its application to antistatic fibre. Sci. Rep..

[34] Davachi S.M., Kaffashi B. (2015). Preparation and Characterization of Poly L-Lactide/Triclosan Nanoparticles for Specific Antibacterial and Medical Applications. Int. J. Polym. Mater. Polym. Biomater..

[35] (2018). Standard Test Method for Deflection Temperature of Plastics Under Flexural Load in the Edgewise Position.

[36] Oksman K., Skrifvars M., Selin J.-F. (2003). Natural fibres as reinforcement in polylactic acid (PLA) composites. Compos. Sci. Technol..

[37] Sitticharoen W., Uthiyoung C., Passadee N., Wongprom C. (2018). Surface treated bagasse fiber ash on rheological, mechanical properties of PLA/BFA biocomposites. Polímeros.

[38] Agunsoye J.O., Aigbodion V.S. (2013). Bagasse filled recycled polyethylene bio-composites: Morphological and mechanical properties study. Results Phys..

[39] Chun K.S., Husseinsyah S., Osman H. (2012). Mechanical and thermal properties of coconut shell powder filled polylactic acid biocomposites: Effects of the filler content and silane coupling agent. J. Polym. Res..

[40] Duan J., Wu H., Fu W., Hao M. (2018). Mechanical properties of hybrid sisal/coir fibers reinforced polylactide biocomposites. Polym. Compos..

[41] Liang Z., Wu H., Liu R., Wu C. (2021). Preparation of Long Sisal Fiber-Reinforced Polylactic Acid Biocomposites with Highly Improved Mechanical Performance. Polymers.

[42] Petinakis E., Liu X., Yu L., Way C., Sangwan P., Dean K., Bateman S., Edward G. (2010). Biodegradation and thermal decomposition of poly(lactic acid)-based materials reinforced by hydrophilic fillers. Polym. Degrad. Stab..

[43] Jiang L., Shen T., Xu P., Zhao X., Li X., Dong W., Ma P., Chen M. (2016). Crystallization modification of poly(lactide) by using nucleating agents and stereocomplexation. e-Polymers.

[44] Suryanegara L., Nakagaito A.N., Yano H. (2010). Thermo-mechanical properties of microfibrillated cellulose-reinforced partially crystallized PLA composites. Cellulose.

[45] Shih Y.-F., Huang C.-C. (2011). Polylactic acid (PLA)/banana fiber (BF) biodegradable green composites. J. Polym. Res..

[46] Aliotta L., Sciara L.M., Cinelli P., Canesi I., Lazzeri A. (2022). Improvement of the PLA Crystallinity and Heat Distortion Temperature Optimizing the Content of Nucleating Agents and the Injection Molding Cycle Time. Polymers.

